# Barriers to mental health service utilization among African immigrants in the United Kingdom: A systematic review

**DOI:** 10.1002/puh2.181

**Published:** 2024-05-19

**Authors:** Archibong Bassey, Rachael Zaka

**Affiliations:** ^1^ Warwick Medical School University of Warwick Coventry UK

**Keywords:** African immigrant, barrier, mental health service, mental health service utilization, systematic review

## Abstract

**Background:**

African immigrants in the United Kingdom (UK) face unique challenges in accessing mental health services (MHSs), in contrast to their peers born in the UK, contributing to their worsening mental and physical health outcomes. This study aims to uncover barriers to MHS utilization and proffer evidence‐based recommendations toward addressing the mental health needs of African immigrants residing in the UK.

**Methods:**

A systematic literature search was conducted across six databases: Medline, PsycINFO, Web of Science, CIHNAL, Scopus, and Embase published up to September 2022. To assess methodological quality of the included studies, the Critical Appraisal Skills Program qualitative checklist and the Mixed Methods Appraisal Tool Version 2018 were used. Consecutively, a deductive thematic analysis was employed to group related barriers within overarching themes.

**Results:**

The study selection process yielded eight studies conducted in the UK, encompassing African populations identifying as African/Afro‐Caribbean origin, Somali refugees, and ethnic minorities of Black/British descent. The findings highlight the complex interplay of key barriers such as stigma, residential instability, cultural influences, discrimination, and accessibility issues, among others, that continue to hinder African populations from accessing and utilizing MHSs. These barriers were categorized into predisposing,enabling and need factors (themes), aligning with Anderson's model of health service utilization, reflecting a comprehensive range of challenges affecting this population.

**Conclusion:**

This systematic review illuminates the myriad barriers faced by African immigrants in utilizing MHSs in the UK, underlining the urgent need for targeted interventions. The findings advocate for the development of culturally sensitive, affordable, and accessible MHSs and policies that address the personal, sociocultural, and structural barriers identified. Collaboration across key stakeholders is highly recommended for advancing equitable and inclusive MHSs for all.

## Graphical abstract

This systematic review reveals that migration brings challenges to mental health, resulting in critical barriers affecting service utilization and worse health outcomes among African immigrants who migrate to the UK. This review highlights gaps in the literature on this issue and the marginalization of African immigrants in such contexts.

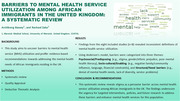

## BACKGROUND

In the 21st century, mental health has risen to a public health issue of concern, with up‐to‐date research pointing out the rise of mental health disorders across the globe, particularly evidenced during the COVID‐19 pandemic, leaving devastating effects on physical health and well‐being among populations [1].

Globally, mental health problems (major depression, obsessive‐compulsive disorders, schizophrenia, alcohol use, and bipolar disorders) are increasing over the years and are among the top 10 leading causes of disability in the world [2]. To achieve universal health coverage for mental health services (MHS), improving service utilization should not only focus on the poorest countries but also include high‐income countries [3]. In high‐income countries, such as the United Kingdom (UK), the cost of mental health problems amounts to over £105.2 billion annually, leading as the number one cause of disability across its diversified population. In addition, further statistics reveal that one in four people experience mental health problems in the UK [4].

The UK like every other high‐income nation continues to attract immigrants, who migrate from different countries seeking work, education, refuge, and other opportunities, for a better life in the UK [5]. Recent statistics estimate that international immigrants make up about 14.4% of the UK's population (i.e., 9.5 million people) [6]. African immigrants constitute roughly 5% of these international immigrants [7] and a reasonable proportion of the UK workforce, making significant economic contributions to the country [8]. Data from the survey of mental health and well‐being, conducted in England in the year 2014, revealed that 23% of Africans or Black British people will experience a common mental health problem at any given week in comparison with 15% of White British people [4].

Although migration provides numerous benefits and opportunities, it also brings about a lot of challenges and risks to mental health and well‐being [9]. African immigrants face critical barriers in accessing and utilizing MHSs, leading to worse mental and physical health consequences in contrast to their peers born in the UK [10]. Although there is insufficient evidence that accounts for the experiences of African immigrants regarding MHS utilization in the UK, the available evidence posits that African immigrants tend to have low levels of MHS utilization, despite having high levels of need [11], and that barriers faced by immigrants’ groups differ and may not always be similar [12].

Therefore, this systematic review seeks to collate and synthesize existing evidence pertaining to the barriers hindering MHS utilization among African immigrants in the UK. Additionally, this review seeks to proffer evidence‐based recommendations toward addressing the barriers to MHS utilization among African immigrants in the UK.

## METHODS

This study utilized the PRISMA guidelines [13] to conduct a mixed‐methods systematic review exploring the barriers to MHS utilization among African immigrants in the UK.

### Eligibility criteria

This review uses the Sample, Phenomenon of Interest, Design, Evaluation, and Research type (SPIDER) tool to guide the search strategy and inclusion criteria:

1. Sample or population of interest—This review will include studies where results have been reported for participants who self‐identify or have been categorized as African immigrants in the UK. Due to classification types and insufficient studies, results may also include Black refugees, as well as people from Afro‐Caribbean or Black British backgrounds, with lived experience of utilizing MHSs.
2. The phenomenon of interest—Studies must explore barriers to MHS utilization and mental health access (up until the last search date, 11 September 2022).
3. Design—Questionnaires, surveys, interviews, focus groups, case studies, observational studies.
4. Evaluation—Outcomes including the subjective outcomes (views, perception, and attitudes).
5. Research type—Qualitative, quantitative, and mixed‐methods studies.

### Exclusion criteria

The following exclusion criteria were created by considering the standards for the inclusion criteria.

1. Studies not related to MHSs or MHS utilization.
2. Systematic reviews as well as non‐peer‐reviewed work, for example, dissertations and posters.
3. Articles that did not address African immigrants.
4. Studies with no evaluable data on barriers.

### Search strategy

Through the assistance of the University Librarian, systematic searches were conducted across six academic databases: Medline, PsycINFO, Web of Science, CIHNAL, Scopus, and Embase. The search strategy was based on synonyms of the primary search terms: (1) barrier, (2) MHS utilization, and (3) African immigrants. The search was tailored for each database, and truncations and wildcards were used where appropriate. Limitations were also set to only identify records published in English and studies conducted with humans. Handsearching of reference lists was also employed.

The full database search strategies, including dates of searches and complete numbers of identified studies per database, are provided in the Supporting Information Appendix file.

### Selection process

The search results from across each database were imported into Covidence software, which was used to screen for duplicates. Two reviewers (AB and RZ) independently screened the title and abstract of all the studies and excluded records that did not meet the inclusion criteria. Articles retrieved were then screened by the two reviewers (AB and RZ) for full‐text review, resulting in further exclusion of records based on wrong outcomes, systematic review or non‐peered work, and wrong population. This resulted in studies included in the final systematic review sample for data extraction, analysis, and quality assessment.

### Data extraction

Using the Covidence software, the following relevant data were extracted from each study: author, year of publication, location in the UK in which the study was conducted, study design, sample size, population description, study outcome measured, and summary of findings (barriers). Data extracted were later verified for accuracy.

### Quality assessment

The included studies for the systematic review were a combination of qualitative and mixed‐methods studies. Hence, to critically appraise these studies, two separate standardized tools were employed: the Critical Appraisal Skills Program (CASP) qualitative checklist and the Mixed Methods Appraisal Tool (MMAT) Version 2018 [14], as CASP tools do not include a mixed‐methods checklist [15]. For qualitative studies (*n* = 7), the CASP checklist consisted of 10 questions; the first 2 questions were screening questions and were employed to evaluate the methodological quality. The MMAT version 2018 was used for the single mixed‐method study (*n* = 1) in the current review, as MMAT evaluates five items in total, with two sections scoring each mixed‐method study's qualitative and quantitative approaches.

### Data analysis/synthesis

An evidence table was developed to report the components of each study, using a tailored approach to synthesize the data extracted. Descriptive (narrative) analyses were conducted to analyze and aggregate the included studies. According to the tabulated findings, a deductive thematic synthesis was employed to group‐related barriers within overarching themes [16]. Due to the heterogeneity of the papers, such as in the sample size, population description, reported outcomes, and the age groups of the participants, conducting a meta‐analysis was thought to be impossible.

### Andersen's behavioral model of health services use

Andersen's behavioral model is a multilevel framework that takes into account both individual and contextual factors that affect how people use healthcare services. Andersen [17, 18] stated that the model “… divides the major components of contextual characteristics in the same way as individual characteristics have traditionally been divided—those that predispose …, enable …, or suggest need for individual use of health services.” This brings out the classification of predisposing, enabling, and need characteristics or factors, which has been used to explore MHS use among different populations [19, 20] and even among immigrants and refugees [21]. In tandem with the aim of this review, this model was used to discuss the various barriers arising from the use or nonuse of MHSs among African immigrants residing in the UK.

## RESULTS

### Study selection

**FIGURE 1:**
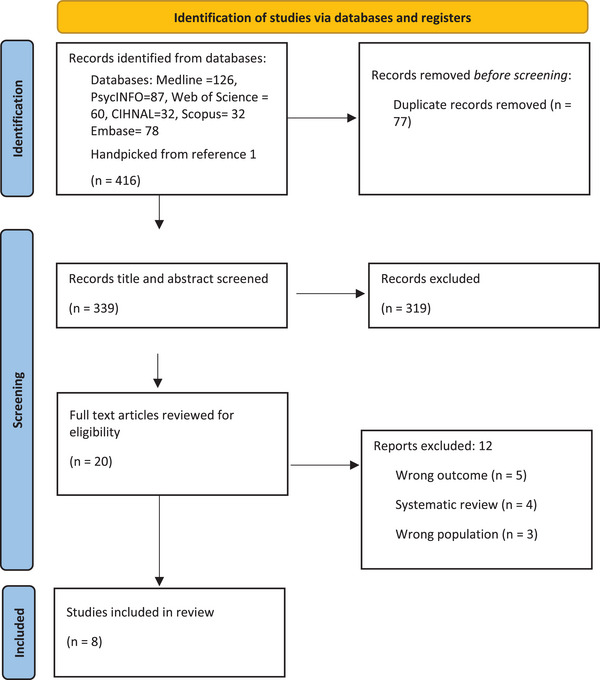
PRISMA flowchart for the selection of the included studies.

Here, the PRISMA guideline [22] was used to develop the flowchart in Figure 1 for the selection of the included studies. The database search (including handpicking of reference) identified 416 published records across 6 databases. These studies were then imported into Covidence. After removing 77 duplicates, 339 studies were screened independently against title and abstracts, resulting in the exclusion of 319 studies that did not address the inclusion criteria. After sorting discrepancies in screening, 20 articles went through the full‐text screening, resulting in the further exclusion of 12 studies based on the following reasons: wrong outcome (*n* = 5), wrong population (*n* = 4), and the article was a systematic review (*n* = 3). This left eight articles in the final sample of the systematic review selected for data extraction, analysis, and quality assessment.

### Study characteristics

The characteristics of the included studies are shown in Table 1. Of the eight studies included in the final systematic review sample, the included studies were all carried out in the UK. However, a majority of these studies were conducted in London (*n* = 5). Other studies were conducted in Aston University, Birmingham (*n* = 1), Southeast England (*n* = 1), and Northwest England (*n* = 1).

**TABLE 1:** Characteristics of the included studies in the systematic review (*n* = 8 studies).

ID	Author/year	Location in the UK in which the study was conducted	Study design and sample size	Population description	Study outcome	Summary of findings (barriers)
1	[23]	East and South London, UK	Qualitative study (*n* = 34)	34 Participants included in the study identified as refugees/migrants from Somalia (An African country) to the UK (13 Somali professionals and 21 Somali lay people aged between 19 and 65 years)	Residential mobility and mental health service utilization	Residential instability Lack of community support Cultural influences, racism and discrimination Changing gender roles
2	[24]	London, UK	Mixed methods (quantitative survey and qualitative focus group discussions) *n* = 189	Out of the 189 Somali respondents for the survey, 143 (76%) were from London, UK (between ages 18 and 65 years). The focus group discussions were held at Queen Mary University of London, the British Refugee Council, and the Somali community settings	Factors that account for poor psychological well‐being and psychiatric disorders among Somali immigrants	Immigration, thwarted aspirations, psychological distress. Unmet expectations, material conditions, changing gender roles and poor mental health Premigration social status, loss of homeland
3	[25]	Islington, London	Cross‐sectional study (interview) (*n* = 1085)	Immigrant elders aged >65 years. Out of 1085 respondents 98 hailed from Africa or Afro‐Caribbean origin	Accessibility to health and social services	Continued stigma Discrimination
4	[26]	Central London, UK	Interpretative phenomenological analysis, Qualitative interviews (*n* = 7)	African Caribbean, women aged between 20s and 50s with lived experiences of emotional distress and accessing mental health services	Explored the perceptions and meanings that the participants gave to their lived experiences of emotional distress and barriers to help seeking	Negative perceptions of mental health services Lack of information regarding access to local Black Caribbean and African mental health services Stigma Social and cultural influences: Being strong and keeping to yourself
5	[27]	Aston University, Birmingham, UK	Qualitative interviews and focus group discussions (*n* = 17)	Afro‐Caribbean undergraduates (18–25 years, 10 females and 7 males) who had lived in the UK for a minimum of 5 years and had experiences of utilizing UK healthcare services the majority of participants identified as Black African (70%)	Barriers and facilitators toward accessing mental health services	Psychological barrier: lack of trust in services, denial about the presence or severity of the problem, fear of non‐confidentiality, fear of exacerbating existing mental health problems Sociocultural barriers: Stigma, the negative influence of family and friends, religion, ethnic/gender prejudice, pride/self‐dependence within the African culture Structural barriers: Negative perceptions toward the possible consequences of accessing mental health services, lack of diversity within mental health services, lack of understanding about mental health and services, practical problems (waiting times, poor quality of service) making services inaccessible.
6	[28]	London	Semi‐structured in‐depth interviews and a semi‐structured questionnaire (*n* = 106)	Members of the Ethiopian community of ages 12 years and above, where majority had experienced difficulties accessing health services	Barriers and enablers which impact the health and well‐being of refugees and should be taken into account in the provision of health and social care services	Stigma of madness Underutilization of mental health facilities Social isolation Language barrier Poor understanding of the system
7	[29]	Southeast England	Qualitative focus group discussions (*n* = 26)	Out of the 26 participants, Black/Black British were *n* = 6 (aged 18 years and above), as about 13 participants did not respond to the question on ethnicity	Factors that influenced access to mental health services	Inability to recognize and accept mental health problems, the positive impact of social networks, stigma against mental health and financial factors Long waiting times, language barriers, inadequate recognition or response to mental health needs, cultural insensitivity, and discrimination toward the needs of BME service users and lack of awareness of different services among service users and providers
8.	[30]	Northwest England	Secondary analysis of qualitative data (*n* = 33)	Vulnerable and hard‐to‐reach group comprising five participants of African‐Caribbean (18% of the total sample size) between the ages of 21 and 80 years	Barriers and facilitators to equitable access to high quality mental health support in primary care	Lack of effective information Multiple stigma(s)

Abbreviations: BME, Black and minority ethnic; UK, United Kingdom.

Most of the study designs were qualitative (*n* = 7), with a single mixed‐methods study (*n* = 1). Table 1 provides a summary of the included papers and an overview of their main findings. Due to the nature of the study, majority reported outcomes for populations identifying as African/Afro‐Caribbean origin (*n* = 4), Somali refugees (*n* = 2), Ethiopia (*n* = 1), and ethnic minority of Black/British backgrounds (*n* = 1).

Data reported on the age of participants varied by age range, as the bulk of the included studies was of people aged 12 years and above. One study focused on elders alone; therefore, they reported age 65 years and above.

Most of the studies involved both males and females (*n* = 7). However, one focused exclusively on females. The number of participants in the included studies varied from 7 to 1085. All studies were conducted between the years 2002 and 2020.

### Risk of bias and quality appraisal

**FIGURE 2:**
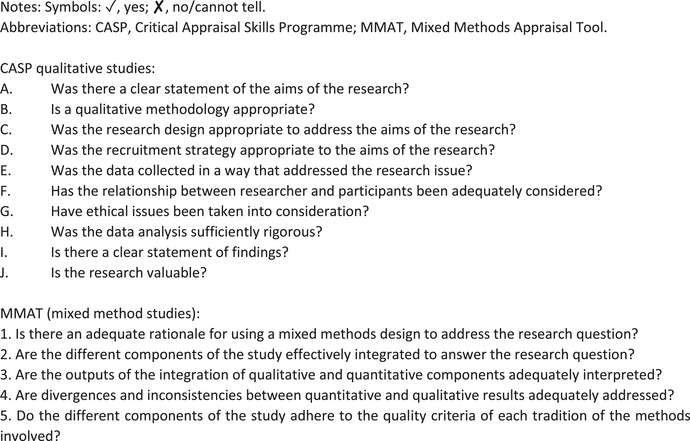
Quality appraisal tools details: Critical Appraisal Skills Program (CASP) and Mixed Methods Appraisal Tool (MMAT).

To appraise the methodological quality of the included articles, the CASP and MMAT tool were readily employed as shown in Figure 2. For qualitative articles, the CASP tool (Table 2) revealed that some articles (*n* = 3) did not explicitly state if the study closely analyzed the researcher's personal role, potential bias, and influence during data collection. One study did not expressly mention whether the ethics committee's approval was sought; however, informed consents were obtained from participants. In summary, the CASP tool highlighted that if the qualitative studies assessed were to be graded, they would be of medium and high quality.

**TABLE 2:** Critical Appraisal Skills Program (CASP) qualitative quality assessment criteria met (*n* = 7 qualitative studies).

Author	A	B	C	D	E	F	G	H	I	J
[23]	✓	✓	✓	✓	✓	✗	✓	✓	✓	✓
[25]	✓	✓	✓	✓	✓	✗	✗	✓	✓	✓
[26]	✓	✓	✓	✓	✓	✓	✓	✓	✓	✓
[27]	✓	✓	✓	✓	✓	✗	✓	✓	✓	✓
[28]	✓	✓	✓	✓	✓	✓	✗	✓	✓	✓
[29]	✓	✓	✓	✓	✓	✓	✓	✓	✓	✓
[30]	✓	✓	✓	✓	✓	✓	✓	✓	✓	✓

Having only one mixed‐method study in the final review, the MMAT tool (Table 3) was employed to critically appraise this. On assessment, the study satisfied all the five criteria of the MMAT, confirming that the article possessed clearly articulated aims, an in‐depth interpretation of data, and different components of the study adhered to the quality criteria of each tradition of both qualitative and quantitative methods involved. It is not recommended to grade the MMAT.

**TABLE 3:** Mixed Methods Appraisal Tool (MMAT) quality assessment criteria met (*n* = 1 mixed‐method study).

Author	1	2	3	4	5
[24]	✓	✓	✓	✓	✓

## STUDY FINDINGS

### MHS utilization among participants

Overall, most of the included studies provided a heterogeneous assessment of MHS utilization among participants, and in some of these studies, it is not clear how MHSs were defined for the purpose of the research and how the rates of MHS utilization were assessed. Of the studies included in the review that assessed MHS utilization, all indicated some type of MHSs accessed by participants [23, 24, 25, 26, 27, 28, 29, 30]. Some articles focused primarily on general healthcare utilization in primary care [25, 28], others investigated service utilization at a psychiatric hospital setting [26], and another looked at mental health prevention through obtaining leaflets containing information about specific voluntary or statutory health and social services [24].

### Barriers to mental health service utilization

**FIGURE 3:**
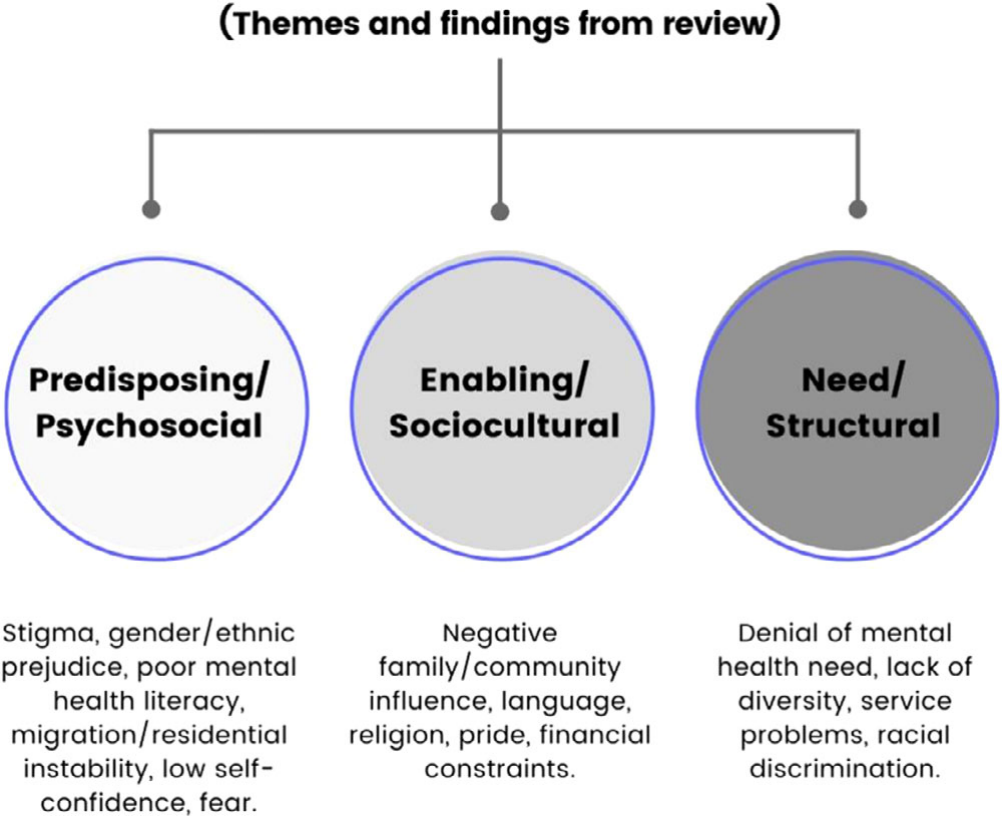
Barriers to mental health service utilization (themes and review findings illustrated).

Three significant barrier themes emerged from a comprehensive thematic analysis of the barriers that were extracted from the included studies: predisposing,enabling and need factors. These can be found in Figure 3 and Table 4.

**TABLE 4:** Barriers to mental health service utilization among African immigrants in the United Kingdom (UK), grouped by themes based on Andersen's behavioral model of health service use.

Barriers	Studies	*n*
**Predisposing factors (personal/psychosocial barriers)**		
Stigma	[23, 24, 25, 26, 27, 28, 29, 30]	8
Lack of knowledge and understanding about mental health and services	[26, 27, 28, 30]	4
Lack of trust in services/fear of non‐confidentiality	[27, 29]	2
Fear of exacerbating existing mental health problems	[27]	1
Lack of self‐confidence	[26, 29, 30]	3
Migration/residential instability	[23, 24]	2
Ethnic and gender prejudice	[23, 24, 26, 27, 29]	5
**Enabling factors (sociocultural barriers)**		
The negative influence of family and friends/lack of community support	[23, 25, 26, 27, 28, 29]	6
Religion (coping mechanism in place of utilizing MHS)	[26, 27]	3
pride/self‐dependence within the African culture	[27]	1
Language	[23, 26, 28, 29]	4
Access to finances/material conditions	[24, 26, 29, 30]	4
**Need factors (structural barriers)**		
Racial discrimination	[23, 29]	2
Lack of diversity within mental health service provision	[27, 29]	2
Practical service problems, i.e., waiting times and poor quality of service	[26, 27, 29]	3
Denial about the presence or severity of the problem (silent suffering when mental health needs are increasing)	[23, 24, 27, 28, 29, 30]	6

#### Predisposing/psychosocial factors

Out of the eight studies included in the review, stigma was a recurring theme among all studies, as participants felt views of others may cause them to put off getting treatment or to hide their disease in order to escape the stigma attached to those with mental health issues [23, 24, 25, 26, 27, 28, 29, 30]. Changing gender roles and gender/ethnic prejudice were common barriers to utilizing mental health care in five reported studies [23, 24, 26, 27, 29]. Additionally, four studies reported that there was a lack of knowledge and understanding about available mental health and services [26–28, 30].

Only one study reported that the fear of accessing professional mental health support will exacerbate the existing mental health problem [27], whereas two studies looked at residential instability and migration experiences as barriers to using MHSs in the host country [23, 24]. However, participants in three studies reported lacking the confidence to approach a provider of MHSs [26, 29, 30]. In other instances, participants’ utilization of mental health treatments was hampered by a lack of trust in services and apprehension over confidentiality, according to two studies [27, 29].

#### Enabling factors

Six studies revealed that participants’ perceptions of accessing mental health treatments are hampered by the negative influence of family and friends as well as a lack of community support [23, 25, 26, 27, 28, 29].

Among African immigrants/refugees, religion was identified in two studies as a coping mechanism in place of utilizing MHSs [26, 27]. Only one study found that the African culture fostered a sense of pride or self‐dependence (the need to look strong), which made it difficult for most participants (80%) to use MHSs [27]. Four studies highlighted language barriers [23, 26, 28, 29], and another four studies have identified material factors such as lack of access to finance and transport/service fees inhibiting the utilization of MHSs [23, 26, 29, 30].

#### Need factors

Six studies reported that the inability to recognize mental health needs leading to denial about the presence or severity of the problem was one of the critical psychological barriers affecting participants [22, 23, 26, 27, 28, 29, 30].

In the UK, two of the studies participants reported that they experienced some form of racism/discrimination, on the part of MHS providers [23, 29], whereas another two noted the lack of diversity within MHSs provision [27, 29]. Conclusively, three papers reported longer waiting times and poor quality of service as key barriers hindering MHS use [26, 27, 29].

## DISCUSSION

In this systematic review, 416 journal articles were screened, and 8 articles were selected that contained qualitative information about understandings of barriers to MHS utilization among African immigrants in the UK. Due to the scarcity of research studies available, the researchers included some studies of Black and minority ethnic and Afro‐Caribbean groups. This review's findings demonstrate that there are indeed numerous challenges that African immigrants may encounter while seeking MHSs in the UK [23, 24, 25, 26, 27, 28, 29, 30]. With 90 % (*n* = 7 of 8) of the included studies as qualitative and one mixed‐method study, the barriers to MHS use among migrants of African descent were explored using thematic synthesis, as illustrated by Braun and Clarke [16]. The overarching themes corresponded to Andersen's behavioral model of health services utilization.

### Predisposing factors

Following the later adaptation of the expanded Andersen model, this is now referred to as “psychosocial factors” [20]. Under this theme, these psychosocial factors include attitudes, values, knowledge, social norms, and perceived control that influence decision‐making to utilize or not to utilize MHSs. The findings from the review identified that concerns about stigma, lack of knowledge about MHSs, and lack of self‐confidence toward MHS use were the top identified barriers theme across studies. Stigma as defined by Fink and Tasman [31] is the marginalization and exclusion of people with mental illnesses. Evidence from Corrigan and Watson [32] reveals that stigma has a dual effect: Self‐stigma is the bias that people with mental illness develop toward themselves, whereas public stigma is the broader public's response to those with mental illness. A lack of knowledge about MHSs could also be referred to as mental health illiteracy as supported by some studies [33]. A cross‐sectional study of African immigrants portrayed that indeed only participants with higher specific knowledge about mental health (such as recognition of schizophrenia as a mental illness) were 26% more likely to report willingness to seek help from a mental health professional [34].

According to a review by McCann et al. [35], African migrant groups are less likely to seek mental health care due to self‐stigma of mental illness, mental health illiteracy, internalization of the stigmatization of mental illness, as well as the resulting decreased self‐confidence and self‐efficacy. This finding is consistent with the result of this review as these barriers may hinder seeking access to or utilizing MHSs. Therefore, more programs need to be developed toward reducing stigma and negative beliefs across African migrant groups.

Another prominent barrier was in the aspect of ethnic and gender prejudice, and migration status as the findings portrayed that females are more likely than males to use mental health care. This is in line with evidence from the study by Saasa et al. [36], who illustrated that rates for MHS utilization were higher in females than in males among African immigrants. Additionally, females were also reported to be more vulnerable to migration‐related threats than males, although this may not be completely true due to notions of masculinity such as the belief of “being strong as a man” resulting in poor MHS use [37]. Due to the complex phenomenon that is migration, interventions should be developed to improve access to MHSs for African immigrants [38].

### Enabling factors

The availability of sufficient resources at the community and individual levels is referred to as enabling factors under Andersen's behavioral model of health service utilization [18]. Barriers under this theme constitute sociocultural barriers. A prominent barrier within this theme is the negative influence of family and friends/the lack of community support, which was reported in the studies, as hindering MHS use [23, 25, 26, 27, 28, 29]. Findings from another study revealed that due to stigma stemming from mental health issues, participants may lose their relationships with families and friends within their communities if they share their mental health concerns [39]. Hence, enabling strong community support systems is encouraged in African migrant communities, toward better MHS use.

Language barriers were also identified as a significant barrier in the findings [23, 26, 28, 29]. Consistent with this, two other studies [40, 41] reported that it was difficult for participants to get mental health care when their language was different from that of their service provider. Therefore, the use of an interpreter was deemed appropriate to solve this communication barrier.

Another prominent barrier identified within this theme was the lack of material conditions (financial resources, transportation, and time) [23, 26, 29, 30]. Financial resources (money, insurance), transportation, and time were deemed critical barriers in the current study. This conclusion is similar to the study by Saasa et al. [36], where the participants (African immigrants) may choose not to receive treatment due to the high cost of mental health care, or they may choose to wait until their next trip to Africa to receive care, or they may seek alternative traditional remedies. In the UK, where health care is free, the increasing visa and health surcharge costs may hinder African immigrants from accessing treatment for mental illness. Evidence has also revealed that free bus passes were associated with reductions in depressive symptoms and feelings of loneliness in the UK [42].

### Need factors

This theme is related to how people perceive their mental health status or how others characterize their requirements in terms of their mental health and functionality (e.g., healthcare provider) also termed structural barriers [20]. A prominent barrier identified here was the participants’ inability to recognize mental health needs leading to denial about the presence or severity of the problem. In a related review by Derr [43], the author uncovered the existence of a strong correlation between the usage of MHSs and perceived and diagnosed need, “as those with a psychiatric diagnosis, poor self‐rated mental health, or former exposure to traumatic experiences were more likely than others to use services.”

The current review also reported longer waiting times and poor quality of service as key MHS utilization barriers among African migrants, which was also highlighted as a critical barrier in a recent study by Fauk et al. [39]. The government's implementation of the first waiting time targets for psychiatric services, including talking therapy, as shown in the NHS website may help in part alleviate long wait times for MHSs.

## LIMITATIONS

There are some limitations to the current study. First, the research was insufficient to allow the generalization of the findings to all African immigrants worldwide or in other parts of the UK given the vast diversity in experiences of African immigrants to the UK and the limited geography of the identified studies.

Additionally, the measures and words used to describe MHSs and mental health professionals varied throughout the included studies of the review. Numerous studies lacked definitions for these key terms. The lack of uniform definitions for MHSs and mental health experts may make it difficult to compare the results of the various studies. Future studies should focus on employing uniform nomenclature and broad categories for MHSs and mental health experts.

## IMPLICATIONS FOR PRACTICE, POLICY, AND FUTURE RESEARCH

This study regarding the barriers to utilizing MHSs among African immigrants in the UK can provide a clear direction and foundation for future equitable service provision and professional mental health practice. Furthermore, variables that lead to the high rates of emotional distress among African immigrants must be evaluated, as well as policies that confront unjust structures that facilitate this higher burden of illness. Advocates for better mental health policies are to push for legislation that would cut the cost of health care for people with lower incomes. Increasing awareness of services, broadening nationwide service utilization routes, and lowering the stigma associated with mental illness are highly recommended steps.

Future research should delve into the various uncovering challenges that affect this population subgroup, as African migrants, as they have been marginalized in research and service provision.

## CONCLUSION

This systematic review provides a comprehensive examination of the myriad barriers to mental health utilization experienced among African immigrants in the UK. The study findings portray the complex interplay of personal/psychosocial, sociocultural, and structural factors that hinder access and utilization of MHS among this population. Addressing these barriers requires multifaceted interventions at the individual, community, and policy levels. With stigma as a recurrent pervasive barrier among the included studies, more efforts should be aimed at behavioral interventions to understand the nature and reduce the many forms of stigma toward MHS use. Policy reforms should stem from evidenced‐based measures to enhance affordability and accessibility to services, while maintaining culturally sensitivity in service provision across the UK. Collaboration with African communities, policymakers, researchers, and service providers, among others, is highly recommended toward achieving improved outcomes toward a more equitable and inclusive MHS provision.

## AUTHOR CONTRIBUTIONS

*Conceptualization; data curation; formal analysis; investigation; methodology; project administration; resources; software; validation; writing—original draft; writing—review and editing*: Archibong Bassey. *Formal analysis; investigation; methodology; writing—original draft; writing—review and editing*: Rachael Zaka.

## CONFLICT OF INTEREST STATEMENT

Authors have no conflicts of interest to declare.

## FUNDING INFORMATION

The authors received no specific funding for this work.

## ETHICS STATEMENT

Ethical approval was not required due to the nature of the research.

## Supporting information

Supporting Information

## Data Availability

Data sharing not applicable.
