# Supplementary material for: Barriers to mental health service utilization among African immigrants in the United Kingdom: A systematic review
Source: Public Health Chall. 2024 May 19;3(2):e181. doi: 10.1002/puh2.181 (PMC12039616; doi:10.1002/puh2.181)
Supplement: Supplementary file 1 — Supporting Information [file PUH2-3-e181-s001.docx]

**Appendix A**

| Ovid MEDLINE(R) ALL <1946 to September 11, 2022> |  |  |
| --- | --- | --- |
|  |  |  |
| 1 | barrier*.mp. | 386164 |
| 2 | obstacle*.mp. | 59895 |
| 3 | hinderance*.mp. | 181 |
| 4 | impediment*.mp. | 10128 |
| 5 | hurdle*.mp. | 15527 |
| 6 | 1 or 2 or 3 or 4 or 5 | 462533 |
| 7 | mental health/ or mental disorders/ | 221329 |
| 8 | Mental Health Services/ or Community Mental Health Services/ | 55896 |
| 9 | 7 or 8 | 254698 |
| 10 | Health Services Accessibility/ or Access*.mp. or utili?ation.mp. | 952490 |
| 11 | "Emigrants and Immigrants"/ or Blacks/ or african immigrant*.mp. | 54473 |
| 12 | 6 and 9 and 10 and 11 | 106 |
| 13 | 6 and 9 and 10 | 3064 |
| 14 | (africa* and immigra*).mp. [mp=title, book title, abstract, original title, name of substance word, subject heading word, floating sub-heading word, keyword heading word, organism supplementary concept word, protocol supplementary concept word, rare disease supplementary concept word, unique identifier, synonyms] | 6722 |
| 15 | 13 and 14 | 8 |
| 16 | exp South Africa/ or exp "Africa South of the Sahara"/ or exp Africa, Central/ or exp Africa, Northern/ or africa.mp. or exp Africa, Southern/ or exp Africa, Eastern/ or exp Africa/ or exp Africa, Western/ | 363634 |
| 17 | immigration.mp. or exp "Emigration and Immigration"/ | 34924 |
| 18 | immigra*.mp. | 59656 |
| 19 | 17 or 18 | 59656 |
| 20 | 16 and 19 | 6561 |
| 21 | 13 and 20 | 126 |

**Appendix B**

| APA PsycInfo <1806 to September Week 2 2022> |  |  |
| --- | --- | --- |
|  |  |  |
| 1 | barrier*.mp. | 90510 |
| 2 | obstacle*.mp. | 22032 |
| 3 | hinderance*.mp. | 33 |
| 4 | impediment*.mp. | 4000 |
| 5 | hurdle*.mp. | 2244 |
| 6 | 1 or 2 or 3 or 4 or 5 | 115194 |
| 7 | mental health.mp. or exp Mental Health/ | 266127 |
| 8 | mental disorders.mp. or exp Mental Disorders/ | 999478 |
| 9 | mental health services.mp. or exp Mental Health Services/ | 71837 |
| 10 | community mental health services.mp. or exp Community Mental Health Services/ | 16019 |
| 11 | 7 or 8 or 9 or 10 | 1157578 |
| 12 | exp Health Care Utilization/ or exp Health Care Access/ or health service accessibility.mp. | 24584 |
| 13 | (access* or utili?ation).mp. [mp=title, abstract, heading word, table of contents, key concepts, original title, tests & measures, mesh word] | 223072 |
| 14 | 12 or 13 | 226593 |
| 15 | 11 and 14 | 60499 |
| 16 | 6 and 15 | 9178 |
| 17 | (africa* and immigra*).mp. [mp=title, abstract, heading word, table of contents, key concepts, original title, tests & measures, mesh word] | 3254 |
| 18 | 16 and 17 | 87 |

| Embase Classic+Embase <1947 to 2022 Week 36> |  |  |
| --- | --- | --- |
|  |  |  |
| 1 | barrier*.mp. | 505748 |
| 2 | obstacle*.mp. | 74773 |
| 3 | hinderance*.mp. | 254 |
| 4 | impediment*.mp. | 12759 |
| 5 | hurdle*.mp. | 19759 |
| 6 | 1 or 2 or 3 or 4 or 5 | 601740 |
| 7 | mental health.mp. or exp mental health/ | 392264 |
| 8 | mental disorders.mp. or exp mental disease/ | 2636222 |
| 9 | mental health services.mp. or exp mental health service/ | 74484 |
| 10 | community mental health services.mp. or exp community mental health service/ | 2046 |
| 11 | 7 or 8 or 9 or 10 | 2808838 |
| 12 | exp health care access/ or health service accessibility.mp. | 90035 |
| 13 | (access* or utili?ation).mp. [mp=title, abstract, heading word, drug trade name, original title, device manufacturer, drug manufacturer, device trade name, keyword heading word, floating subheading word, candidate term word] | 1366046 |
| 14 | 12 or 13 | 1375921 |
| 15 | 11 and 14 | 128786 |
| 16 | 6 and 15 | 13932 |
| 17 | exp "Africa south of the Sahara"/ or exp South Africa/ or exp Africa/ or exp North Africa/ or exp Central Africa/ or africa.mp. | 455426 |
| 18 | exp immigration/ or exp immigrant/ or immigra*.mp. | 51916 |
| 19 | 17 and 18 | 5187 |
| 20 | 16 and 19 | 26 |

**Appendix C**

**Scopus** (Article Title, Abstract, Keywords)

barrier* OR hurdle OR obstacle OR obstruct* OR challenge* OR impediment* OR difficult*

AND "mental health" OR "mental disorders" OR "mental health services" OR "community mental health services"

AND access* OR utili?ation

AND africa* AND immigra*

Last Search conducted 11th September 2022– results: 32
